# Underlying Skills of Oral and Silent Reading Fluency in Chinese: Perspective of Visual Rapid Processing

**DOI:** 10.3389/fpsyg.2016.02082

**Published:** 2017-01-10

**Authors:** Jing Zhao, Rosa K. W. Kwok, Menglian Liu, Hanlong Liu, Chen Huang

**Affiliations:** ^1^Key Laboratory of Learning and Cognition, Department of Psychology, College of Education, Capital Normal UniversityBeijing, China; ^2^Beijing Advanced Innovation Center for Imaging Technology, Capital Normal UniversityBeijing, China; ^3^Centre for Research in Psychology, Behaviour and Achievement, Department of Psychology, Coventry UniversityCoventry, UK

**Keywords:** visual rapid temporal processing, visual rapid simultaneous processing, visual attention span, Chinese reading fluency, silent reading, oral reading

## Abstract

Reading fluency is a critical skill to improve the quality of our daily life and working efficiency. The majority of previous studies focused on oral reading fluency rather than silent reading fluency, which is a much more dominant reading mode that is used in middle and high school and for leisure reading. It is still unclear whether the oral and silent reading fluency involved the same underlying skills. To address this issue, the present study examined the relationship between the visual rapid processing and Chinese reading fluency in different modes. Fifty-eight undergraduate students took part in the experiment. The phantom contour paradigm and the visual 1-back task were adopted to measure the visual rapid temporal and simultaneous processing respectively. These two tasks reflected the temporal and spatial dimensions of visual rapid processing separately. We recorded the temporal threshold in the phantom contour task, as well as reaction time and accuracy in the visual 1-back task. Reading fluency was measured in both single-character and sentence levels. Fluent reading of single characters was assessed with a paper-and-pencil lexical decision task, and a sentence verification task was developed to examine reading fluency on a sentence level. The reading fluency test in each level was conducted twice (i.e., oral reading and silent reading). Reading speed and accuracy were recorded. The correlation analysis showed that the temporal threshold in the phantom contour task did not correlate with the scores of the reading fluency tests. Although, the reaction time in visual 1-back task correlated with the reading speed of both oral and silent reading fluency, the comparison of the correlation coefficients revealed a closer relationship between the visual rapid simultaneous processing and silent reading. Furthermore, the visual rapid simultaneous processing exhibited a significant contribution to reading fluency in silent mode but not in oral reading mode. These findings suggest that the underlying mechanism between oral and silent reading fluency is different at the beginning of the basic visual coding. The current results also might reveal a potential modulation of the language characteristics of Chinese on the relationship between visual rapid processing and reading fluency.

## Introduction

Reading fluency refers to reading rapidly and accurately to comprehend the text (Langer et al., 2013). One of the most common symptoms for developmental dyslexia is a persistent failure to develop fluent reading skills, which can have severe academic, economic, and psychosocial consequences (Fraga González et al., 2015). Hence, it is necessary to explore the underlying mechanism of reading fluency in order to help these struggling readers to bring up their comprehension skills. The majority of relevant studies have focused on oral reading fluency which is the primary reading mode (Kim et al., 2011; van den Boer et al., 2014). However, much less is known on silent reading fluency, which is the more common mode of reading (van den Boer et al., 2014). Some researchers suggested that silent reading and oral reading may essentially involve the same processes, except that there was the addition of articulatory demands for oral reading (Ashby et al., 2012). In contrast, other studies found that oral and silent reading differed in their cognitive mechanisms. For example, the main goal in oral reading is to pronounce every word and the comprehension of text is the secondary goal. This process focuses more on the grapheme-to-phoneme rules in the sublexical route. On the other hand, the main goal in silent reading is to comprehend and assimilate the meaning of the text which relies on the grapheme-to-semantic decoding in the lexical route (Galin et al., 1992; Snellings et al., 2009; van den Boer et al., 2014). The above studies compared oral and silent reading fluency from the linguistic aspect. However, it has been reported that it is difficult to ameliorate the reading fluency deficit for the dyslexics by providing an intervention that focuses on language skill only (Langer et al., 2013). Therefore, it might be informative to investigate the underlying skills that supports reading fluency development. Given that reading is a sensory process that involves graphic input, the letters and words on the page, it is logical to trace back to the visual perception to understand the reading process (Rauschecker et al., 2011; Grainger et al., 2012; Ziegler et al., 2013). The present study aims to address the following two research questions, (1) what is the role of general perceptual processing in reading fluency, and (2) whether the mechanisms of oral and silent reading fluency are comparable at the basic visual level?

Previous studies reported that visual rapid processing played an important role in reading fluency (McLean et al., 2011; Lobier et al., 2012, 2013; Main et al., 2014). Visual rapid processing includes rapid temporal processing and rapid simultaneous processing (Lallier and Valdois, 2010). The visual rapid temporal processing refers to the sequential dimension of processing, i.e., discriminating the succession of two or more stimuli (Farmer and Klein, 1995; Lallier and Valdois, 2010). Relevant research usually adopted tasks of gap detection, temporal order judgment, coherent motion detection, moving/flickering grating detection to measure the ability of visual rapid temporal processing (Farmer and Klein, 1995; McLean et al., 2011). The visual rapid simultaneous processing refers to the spatial dimension of processing, i.e., integrating multiple stimuli in parallel (Lallier and Valdois, 2010). The visual attention span is usually considered as an index of the visual rapid simultaneous processing (Bosse et al., 2007; Lallier and Valdois, 2010). Studies in alphabetic languages have found that children’s performance in coherent motion detection, temporal order judgment, and moving grating detection was significantly correlated with their scores in oral reading fluency test. This illustrates the relationship between the visual rapid temporal processing and oral reading fluency (word level: Kevan and Pammer, 2008; van Zuijen et al., 2012; Main et al., 2014; sentence level: Demb et al., 1997; Ben-Shachar et al., 2007; Lawton, 2011). Yet, no correlation was observed between visual rapid temporal processing and reading fluency in silent mode (Steinbrink et al., 2014).

Moreover, it has been found that the visual attention span was correlated with the scores of reading fluency test in both oral (Bosse et al., 2007; Lobier et al., 2012; Germano et al., 2014; Tobia and Marzocchi, 2014) and silent modes (van den Boer et al., 2014). van den Boer et al. (2014) conducted a direct comparison between silent and oral reading fluency. They found that the visual attention span was correlated equally with both oral and silent reading modes but it only made a significant unique contribution to silent reading. The above findings seemingly suggested that the visual rapid temporal processing was more remarkably related to oral reading fluency, and visual rapid simultaneous processing was more closely associated with silent reading fluency. It has been suggested that visual rapid temporal processing may play a role in the preattentive control of spatial selection (i.e., position encoding, Vidyasagar, 2005; Pammer and Kevan, 2007). The letter-by-letter spelling in oral reading fluency involved the position encoding (Wolf and Katzir-Cohen, 2001), and thus there was a close relationship between visual rapid temporal processing and oral reading. Silent reading was implicated with visuospatial processing and parallel processing of multiple orthographic units (van den Boer et al., 2014), which can explain the relationship between visual rapid simultaneous processing and silent reading. And this implies that the underlying mechanisms of oral and silent reading fluency might be different from the aspect of basic visual processing.

Most of the above studies has utilized the coherent motion detection and temporal order judgment tasks to measure visual rapid temporal processing. However, some researchers indicated that the temporal order judgment task might involve the processing of memory (Edwards et al., 2004), and both the temporal and spatial dimensions of visual rapid processing might be incorporated in the coherent motion detection task (McLean et al., 2011). The “phantom contour paradigm” designed by Sperling et al. (2003) can be adopted to measure the ability of visual rapid temporal processing and it diminished the processing of memory in the task. McLean et al. (2011) used this paradigm and found that the children’s scores in this visual processing test were correlated with their oral reading fluency of sentences, suggesting the relationship between visual rapid temporal processing and oral reading fluency. However, as far as we are aware, no research has attempted to use the phantom contour paradigm to investigate the relationship between oral/silent reading fluency and visual rapid temporal processing.

Additionally, the aforementioned studies on visual attention span usually used a letter-report task. This has two potential caveats: it requires a verbal response and the usage of verbal stimuli. As a result, this task may tap into the visual to phonological mapping rather than visual rapid simultaneous processing. In order to separate these two processes, it is necessary to use a parallel visual processing task with non-verbal stimuli and no verbal report. The visual 1-back task in Lallier et al. (2015) meets this requirement and the task will be explained further in the Method session. In addition, the participants in the aforementioned studies were all developing readers. Their proficiency in oral and silent reading might differ and this may influence the correlational relationship between the two reading modes. In the current study, we recruited skilled readers to ensure that participants are equally proficient in both oral and silent reading.

To our knowledge, all the relevant studies on skilled readers only examined oral reading fluency, and there were mixed results with respect to the relationship between reading fluency and visual rapid processing. For instance, Amitay et al. (2002) did not find a significant correlation between visual rapid temporal processing and oral reading fluency in Hebrew adults. However, this relation was reported in English adults (Main et al., 2014). The inconsistent findings might be due to the difference in orthographic depth. English is considered to have a deeper orthography than Hebrew (Seymour et al., 2003). The acquisition of the grapheme-to-phoneme correspondence (GPC) rule is easier in languages with shallow orthography (e.g., Hebrew) as compared to languages with deep orthography (e.g., English; Kwok et al., 2016). As the result, the automatization of the orthographic-to-phonological mapping would be achieved earlier in languages with shallow orthography (Wolf and Katzir-Cohen, 2001; Xue et al., 2013). The efficient orthographic-to-phonological mapping is critical for reading fluency (especially for oral reading fluency, Norton and Wolf, 2012; Eberhard-Moscicka et al., 2014; Hakvoort et al., 2015), and it has been suggested that the visual rapid temporal processing played a role in the mapping between spelling and sound which required the visually serial engagement and disengagement from each sub-lexical unit (Gori et al., 2014; Ruffino et al., 2014). Accordingly, due to its’ consistent grapheme-phoneme correspondence in shallow languages, the skilled readers may not show a close relationship between the (oral) reading fluency and visual rapid temporal processing in shallow orthographies. In contrast, the automatization in the orthographic-to-phonological mapping is inconsistent in languages with deep orthography, and therefore potentially visual rapid temporal processing may have an impact on the oral reading fluency. As to the visual rapid simultaneous processing in skilled readers, Awadh et al. (2016) recruited Arabic, French, and Spanish adults. They found that only the visual attention span of French adults was correlated with the scores of oral reading fluency test. This revealed the relationship between visual rapid simultaneous processing and oral reading fluency in a deep orthography. The authors pointed out that the inconsistent findings between the relationship of visual rapid processing and oral reading fluency may be modulated by the transparency of the languages (Awadh et al., 2016).

In contrast to alphabetic languages, Chinese has a logographic writing system. The visual configuration of a Chinese character is complex, and is markedly different from that of an alphabetically-written word. It has been suggested that visual processing played a more important role in Chinese reading (McBride-Chang et al., 2011). Previous studies have indicated that the visual perceptual processing (e.g., the low-spatial-frequency sensitivity, geometric-figure processing) had an impact on the recognition and encoding processes during Chinese reading in typically developed children and adults (Luo et al., 2013; Yang et al., 2013; Zhao et al., 2013, 2014). Various studies have shown that Chinese individuals with developmental dyslexia exhibit visual deficits (Chung et al., 2008; Wang et al., 2010; Meng et al., 2011; Qian and Bi, 2014), and visual function training for dyslexics can improve their reading-related skills (Meng et al., 2014; Wang et al., 2014; Qian and Bi, 2015).

Moreover, Chinese does not have GPC rules; it has instead a logographic writing system. The mapping between visual form of a Chinese character and its speech sound is based on a globally addressed way, that is, the orthography of a whole character is linked to its pronunciation; in contrast, it is an assembled way following the GPC rules in alphabetic languages (Tan et al., 2005). Then what is the relationship between visual rapid processing and reading fluency in Chinese? Whether this relation would be in line with the prediction based on findings in alphabetic languages, that is, the relationship would be significant in the language without GPC rules? Or the relationship between visual rapid processing and Chinese reading fluency would be affected by the characteristic of Chinese orthographic-to-phonological mapping, revealing the modulation of Chinese specificity? Theoretical interest lay in the relationship between visual rapid temporal/simultaneous processing and reading fluency of different modes (oral and silent) in Chinese. This can help to elucidate the role of orthographic consistency in the relationship of visual rapid processing and reading fluency. Previous studies on children indicated that their performance in the coherent motion detection and phantom contour tasks was related to their oral reading fluency (single character level: Qian and Bi, 2014; Xiao et al., 2014; Qian et al., 2015) but not silent reading fluency (Meng et al., 2011). These findings implied that the visual rapid temporal processing may be more closely associated with oral reading fluency in Chinese. This result was consistent with the findings in alphabetic languages (Main et al., 2014; Steinbrink et al., 2014). Yet, these studies did not compare visual processing in oral and silent reading directly and they had recruited developing readers as their participants.

Thus far, there has been only one relevant study that investigated the relationship between visual processing and reading in skilled readers (Qian et al., 2015). Qian et al. (2015) found that the adults’ brain activation induced by the coherent motion detect task was correlated with their scores of oral reading fluency test of the Chinese characters, suggesting that the visual rapid temporal processing of Chinese skilled readers was related to their oral reading fluency. There has been no relevant report that explore the relationship between visual rapid simultaneous processing and reading fluency. Moreover, visual attention span has been found to relate to visuospatial and global processing when participants had to process multiple units (Xue et al., 2013). Given that global visual processing plays an important role in Chinese reading (Wang et al., 2014), it is necessary to explore the relationship between the visual rapid simultaneous processing and reading fluency in Chinese.

The present study aimed to examine the relationship between visual rapid temporal/simultaneous processing and reading fluency (oral and silent modes) in Chinese skilled readers. This helps to explore the role of orthographic consistency in this relation and to compare the underlying mechanisms of oral and silent reading fluency from the perspective of general perceptual processing. Fluent reading occurs at various levels of language process (Wolf and Katzir-Cohen, 2001; Kim et al., 2011) where the visual rapid processing might play different roles (Liu et al., 2015). For the single-character level, there are a large number of visually similar characters in Chinese (e.g., 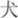 /tai4/, meaning very—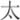 /quan3/, meaning dog), and the ability to process detailed visual information quickly is critical for reading Chinese characters as it enables children to effectively map the Chinese orthography onto semantics and phonology. For the sentence level, there is no interword spacing for multicharacter words in Chinese, and effective visual processing may allow the reader to focus on a target character rapidly, with reducing the crowding effect. Therefore, the present study systematically tested the reading fluency from both the single character and sentence levels. Furthermore, the phantom contour paradigm of Sperling et al. (2003) and visual 1-back task of Lallier et al. (2015) were adopted to measure the visual rapid temporal and simultaneous processing, respectively. Based on previous studies (van den Boer et al., 2014; Qian et al., 2015; Awadh et al., 2016), we make the following two predictions:

(1) The visual rapid temporal processing of the Chinese skilled readers might be more related to oral reading fluency as compared to silent reading;
(2) Chinese skilled readers’ visual rapid simultaneous processing would be associated with silent reading fluency more remarkably than oral reading fluency.

## Materials and Methods

### Participants

A total of 60 undergraduate and graduate students in Beijing participated in the present study. The data from two participants were excluded from the final analysis because they did not seriously complete the visual 1-back task. The remaining 58 participants, who ranged in age from 19 to 25 years, with a mean of 23 years. All of the participants were right-handed Mandarin speakers, and had normal hearing and normal or corrected-to-normal vision without ophthalmological or neurological abnormalities. Written consent was obtained from each participant prior to the experiment. The study was approved by the institutional review board of the Department of Psychology, Capital Normal University.

### Procedure

All participants were tested individually in a quiet room. At the beginning of the experiment, the experimenter explained the procedure in detail from a standard script. Two reading tasks were administered to measure reading fluency of single characters and sentences respectively. Within each level of one reading fluency test, participants performed the same task in both the oral and silent reading conditions so as to reduce the influence from differences in experimental tasks and reading materials on the comparison between the oral and silent reading. The oral and silent reading conditions for each reading fluency test were separated by the tests of visual rapid processing in order to diminish the influence of practice effect. Consequently, there were three sessions in the present study. In the first and third sessions, the reading fluency tests of both single-character and sentence levels were conducted, in which the reading modes between the two sessions were reversed. For example, if the first session included an oral test of character reading fluency and a silent test of sentence reading fluency, then a silent test of character reading fluency and an oral test of sentence reading fluency were conducted in the third session. There were four patterns for the implementation order of the reading tests, and accordingly participants were randomly and equally divided into four groups: (1) the first session— orally reading single characters and sentences, the third session— silently reading single characters and sentences; (2) the first session— orally reading single characters and silently reading sentences, the third session— silently reading single characters and orally reading sentences; (3) the first session— silently reading single characters and orally reading sentences, the third session— orally reading single characters and silently reading sentences; (4) the first session— silently reading single characters and sentences, the third session— orally reading single characters and sentences. In the second session, the two tests of visual rapid processing were administered. Within each session, the order of the tests was random. There was a 1-min rest between successive sessions.

### Measurements

#### Reading Fluency Tests

##### Single-character level

Reading of single characters was assessed with a paper-and-pencil lexical decision task. Children were presented with a list of 400 Chinese characters intermixed with 13 non-characters. The split-half reliability was 0.93. Participants were required to read the items either aloud or silently and to cross out the non-characters, with the time limit of 1 min. At the end of this test, participants were asked to mark the last item they read. The score consisted of the number of items read minus the number of errors, in which errors were non-characters that were not identified as well as real character that were incorrectly crossed out.

##### Sentence level

A sentence verification task was developed to assess reading fluency in sentence level. The split-half reliability was 0.85. A total of 54 sentences were constructed (four for the practice session and the rest of the 50 sentences were used in the formal test). The sentences were all about simple facts and the length of each sentence varies from seven to twenty-two characters (e.g., “
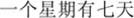
” means that “There are 7 days in a week”). Half of the sentences were true and the other half were false. This test was presented by a Dell laptop. Participants were seated approximately 50 cm from a computer screen. Within each trial, a fixation point displayed in the center of the screen for 500 ms, and then a target of a complete sentence appeared. Participants were instructed to read the sentence as accurately and quickly as possible either aloud or silently, and to press the space bar once finishing reading the sentence. The interval between the beginning of the sentence presentation and the time of pressing the space bar was recorded. The reading speed for one sentence was calculated based on the relative ratio of the number of Chinese characters in the sentence to the interval of reading this sentence, and the mean of reading speed was computed. After pressing the space bar, a judgment was followed, in which participants were require to press different keys to judge the veracity of the sentence, with “f” for false and “j” for true. The accuracy for the veracity judgment was recorded.

#### Tests of Visual Rapid Processing

##### Visual rapid temporal processing

**FIGURE 1:**
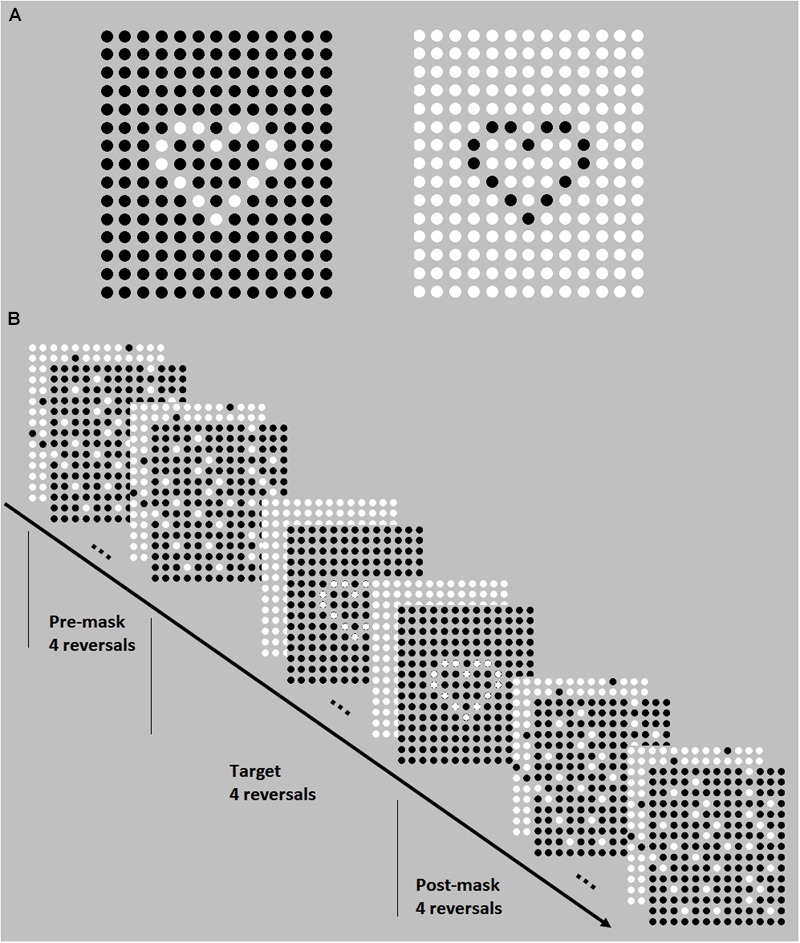
**The targets (A)** and the presentation format of each trial **(B)** in the phantom contour paradigm. The targets are two images of heart consisted of phase-reversing dots, which were alternately presented with a phantom contour of heart appearing. Each trial followed the presentation format as below **(B)**: mask (four reversals), target (four reversals), mask (four reversals). And then the participants were required to press different keys to judge whether there was a phantom contour of heart or not.

Based on previous studies (Sperling et al., 2003, 2006; Xiao et al., 2014), the phantom contour paradigm was adopted to measure participants’ visual rapid temporal processing. The test–retest reliability was 0.78. This test was conducted by a Dell laptop, and its display resolution was set at 1024 × 768 with the monitor refresh rate of 75 Hz. Two images of heart consisted of phase-reversing dots were used as targets in the present study (**Figure 1A**), which were alternately presented with a phantom contour of heart appearing. A brief mask preceded and followed the presentation of the target shape. The mask was consisted of a random assortment of phase-reversing dots. Each trial followed the presentation format as below (**Figure 1B**): mask (four reversals), target (four reversals), mask (four reversals). And then the participants were required to press different keys to judge whether there was a phantom contour of heart or not, with “v” for yes and “b” for no. The probability of an absence of the heart shape was 20%. Referring to the relevant literature (Levitt, 1971; Sperling et al., 2003, 2006), a two-up/one-down staircase was used to measure the reversal rate at which the participant could perform the task at 70.7% correct. Details of the two-up/one-down staircase was as below: after two consecutive correct trials, the duration of each image frame would decrease by the relevant step size; and after every single incorrect trial, the duration of each image frame increased by the relevant step size. The staircase procedure terminated after 15 reversals. The minimum value of the duration of each image frame was the monitor refresh time, which corresponding with the maximum value of temporal resolution, that is, one frame of the monitor refresh rate (i.e., 75 Hz). According to a pre-study, we set the step sizes thus: three times as much as the monitor refresh time for the first three reversals, twice as much as the monitor refresh time for the 4–9th reversals, and the monitor refresh time for the last six reversals. The average for the last six reversals was taken to estimate the threshold of frame rate. The staircase started from the value above the predictable threshold (about 10.8 Hz), and the presenting procedure of staircase was programmed with E-prime 1.1. In this visual test, participants sat 50 cm away from the monitor. Individual dots subtended an area of approximately 0.6° × 0.6°, and the entire image subtended 13.7° × 11.6°visual angle.

##### Visual rapid simultaneous processing

**FIGURE 2:**
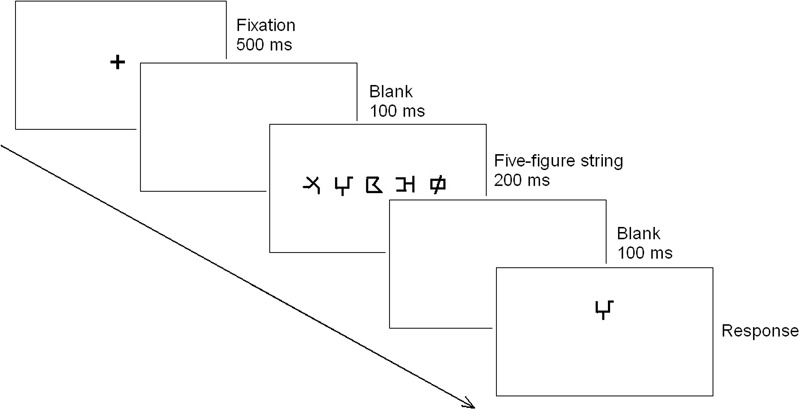
**The presentation format of each trial in the visual 1-back task.** In each trial, a fixation point was firstly presented for 500 ms in the screen center, followed by a white screen of 100 ms and then the five-figure string centered on fixation for 200 ms. The string was followed by a white screen lasting 100 ms and finally a single figure (target) appearing below or above (half of the trials) the median horizontal line. Participants were asked to press different keys to judge whether the target figure was present in the above string or not.

A visual 1-back paradigm (Lallier et al., 2015) was adopted to measure the visual attention span skills (i.e., revealing the ability of visual rapid simultaneous processing with non-verbal stimuli and no verbal response). The test–retest reliability was 0.81. The stimuli in this test were 15 figures. Their visual complexity was evaluated by another 20 undergraduate (12 females) who did not participate the formal experiment. The results of the rating scale with six points (one = The figure is not complex at all, six = The figure is extremely complex) showed the mean value of the visual complexity is 2.27, and the rating of each figure is below three point. A list of 120 five-figure strings was created using the 15 figures. Each string did not include the same figure twice. They were presented in black on a white screen with E-prime 1.1 on a Dell laptop. The display resolution was set at 1024 × 768 with the monitor refresh rate of 75 Hz. The visual angle of the strings were 7.9° × 0.8° at a distance of 50 cm. The center-to-center distance between each adjacent figure was 1.7°. In each trial (**Figure 2**), a fixation point was firstly presented for 500 ms in the screen center, followed by a white screen of 100 ms and then the five-figure string centered on fixation for 200 ms. The string was followed by a white screen lasting 100 ms and finally a single figure (target) appearing below or above (half of the trials) the median horizontal line. Participants were asked to press “z” as quickly and accurately as possible when the target figure was present in the above string and to press “b” when it was absent. The target figure was replaced by a blank screen after the response. The blank screen was displayed in a random interval (from 1000 to 1500 ms) between successive trials. The 120 trials were presented randomly, and included 75 target-present trials (the 15 figures were presented five times as targets, once at each position in the string) and 45 target-absent trials (the 15 figures were presented three times as target). The test trials were preceded by 10 practice trials. The means of response time and accuracy were recorded.

## Results

Firstly, a statistical power analysis was conducted by using the software of G^∗^Power Version 3.1.9.2, and the result showed a power of 90.43% with the sample size of 58.

Means and standard deviations of reading fluency and visual measures are presented in **Table 1**.

**Table 1:** Means and standard deviations of reading fluency and visual measures.

Measurements		Scores
**Reading fluency**		
*Single-character level*	Oral reading (c/min)	136.53 (31.90)
	Silent reading (c/min)	214.16 (75.91)
*Sentence level*		
	Oral reading-accuracy	0.92 (0.03)
	Oral reading-speed (c/min)	303.12 (84.43)
	Silent reading-accuracy	0.92 (0.04)
	Silent reading-speed (c/min)	503.21 (188.76)
**Visual rapid processing**		
*Temporal processing*	Temporal threshold (Hz)	31.25 (11.58)
*Simultaneous processing*	Accuracy of visual 1-back task	0.59 (0.08)
	Reaction time of visual 1-back task (ms)	1057.66 (240.47)

*Standard deviations were shown in the parentheses for each item in the “Scores” column. Measure units are in the parentheses for each item in the “Measurements” column, in which the unit of reading speed “c/min” means the number of characters correctly read in 1 min. Hz, hertz; ms, millisecond.*

### The Comparison of Scores between Oral and Silent Reading Fluency Tests

Paired-sample *t*-test were conducted to compare the scores between oral and silent reading tests. In single-character level, the score in silent reading test was significantly higher than that in oral reading test [*t*_57_ = 7.83, *p* < 0.001], i.e., participants correctly read more characters in 1 min for silent reading as compared to oral reading. In sentence level, the accuracy for the veracity judgment was high, which was higher than 0.9 for most participants. And the accuracy between the two reading modes was similar [oral: 0.92; silent: 0.92, *t*_57_ = 0.26, *p* = 0.80], suggesting no significant difference in the task difficulty between the two modes. The reading speed in the sentence reading fluency showed a significant effect of reading mode [*t*_57_ = 7.34, *p* < 0.001], in which silent reading was significantly faster than oral reading.

### Relations between Visual Rapid Processing and Chinese Reading Fluency

A Pearson product-moment correlation was conducted to analyze the relationship between visual rapid processing and reading fluency (**Table 2**).

**Table 2:** Correlation between visual rapid processing and Chinese reading fluency.

	Single character		Sentence			
	Oral	Silent	Oral_acc	Oral_speed	Silent_acc	Silent_speed
Temporal threshold	-0.03 (0.80)	0.02 (0.87)	0.08 (0.59)	-0.15 (0.30)	0.09 (0.54)	0.003 (0.99)
Accuracy of visual 1-back task	0.01 (0.92)	-0.11 (0.42)	-0.16 (0.36)	0.06 (0.70)	-0.02 (0.88)	-0.21 (0.14)
Reaction time of visual 1-back task	0.01 (0.97)	-0.14 (0.29)	0.10 (0.50)	-0.24^+^ (0.09)	0.10 (0.50)	-0.45^∗∗^ (0.001)

*Oral_acc, the accuracy in oral reading fluency in the sentence level. Oral_speed, the reading speed in oral reading fluency in the sentence level, the unit is the number of characters per minute. Silent_acc, the accuracy in silent reading fluency in the sentence level. Silent_speed, the reading speed in silent reading fluency in the sentence level. ^+^*p* < 0.1; ^∗∗^*p* < 0.01.*

The results showed that the threshold of visual rapid temporal processing was correlated with neither oral nor silent reading fluency in single-character or sentence levels (*p* > 0.1 for all).

There was no significant correlation between the accuracy in visual 1-back task and any score of reading fluency tests (*p* > 0.1 for all). The mean reaction time in the test of visual rapid simultaneous processing was negatively correlated with the reading speed in both oral [*r* = -0.29, *p* = 0.09, marginally significant] and silent [*r* = -0.45, *p* = 0.001] modes, where shorter reaction time for visual rapid simultaneous processing corresponded to higher speed of reading. The two correlation coefficients were compared in order to examine whether the visual rapid simultaneous processing was more closely related to silent reading fluency as compared to oral reading fluency. The correlation coefficients were firstly transformed to standardized values through the formula of Fisher (1970),

(1) $$Z_{r}=\frac{1}{2}In(\frac{1+r}{1-r}).$$

Then the relevant standardized values were compared referring to the formula of Snedecor (1980),

(2) $$Z=\frac{Z_{r_{1}}-Z_{r_{2}}}{\sqrt{\frac{1}{\left(n_{1}-3)+(n_{2}-3\right)}}}.$$

Finally, the results showed that the two correlation coefficients were significant differently [*Z* = 1.97, *p* < 0.05], that is, the mean reaction time in visual 1-back task was more closely related to silent reading fluency than to oral reading fluency.

In order to explore the unique contribution of visual rapid simultaneous processing to silent (oral) reading fluency, hierarchical regression analysis was conducted. The temporal threshold in the phantom contour task and the accuracy of visual 1-back task were entered into the regression equation at the first and second steps, and then the mean reaction time in the visual 1-back task was entered at the last step (**Table 3**). And the reading speed in oral and silent reading modes was respectively regarded as the dependent variable. The details of relevant results are displayed in **Table 3**.

**Table 3:** Hierarchical regression estimating the predictive power of visual rapid simultaneous processing on reading fluency.

Dependent variable	Step	Independent variable	Δ*R*^2^	β	*F*-values	*p*
Oral_speed	1	Temporal threshold	0.023	-0.15	1.12	0.30
	2	Accuracy of visual 1-back task	0.004	0.06	0.63	0.54
	3	Reaction time of visual 1-back task	0.057	-0.24	1.38	0.26
Silent_speed	1	Temporal threshold	0.000	0.007	0.00	0.96
	2	Accuracy of visual 1-back task	0.045	-0.208	1.08	0.35
	3	Reaction time of visual 1-back task	0.196	-0.443	4.77^∗∗^	0.006

*Oral_speed, the reading speed in oral reading fluency in the sentence level, the unit is the number of characters per minute. Silent_speed, the reading speed in silent reading fluency in the sentence level. ^∗∗^*p* < 0.01.*

When the speed of orally reading sentence was regarded as the dependent variable, the visual temporal threshold, the accuracy and reaction time of the visual 1-back task could only account for 2.3, 0.4, and 5.7% of the variance in the oral reading speed, respectively (*p*s > 0.05 for all). Especially, although the reaction time of visual 1-back task exhibited a relation to the oral reading speed, this correlation was not maintained in the regression analysis after visual temporal threshold and accuracy of visual 1-back task were removed. When the reading speed in silent mode was treated as the dependent variable, the visual rapid temporal processing had no contribution to silent reading speed, and the accuracy of visual 1-back task only could account for 4.5% of the variance in silent reading speed (*p* > 0.05), whereas the mean reaction time of visual 1-back task could independently account for 19.7% of the variance in the speed of silent reading fluency (*p <* 0.01).

## Discussion

The present study investigates the relationship between visual rapid temporal/simultaneous processing and Chinese reading fluency in oral and silent reading. The results showed that there was no significant correlation between temporal threshold in the phantom contour paradigm and the scores in reading fluency tests, revealing the absence of relationship between visual rapid temporal processing and reading fluency. On the other hand, the reaction time in visual 1-back task was correlated with the reading speed in both oral and silent reading of sentences. This demonstrates the association between visual rapid simultaneous processing and Chinese reading fluency. Most importantly, the visual rapid simultaneous processing exhibited a significant unique contribution to reading fluency in silent mode but not in oral reading fluency. These findings suggested that the cognitive mechanisms in silent and oral reading fluency might be different in the basic level of visual coding.

In the current results, visual rapid temporal processing did not show a significant correlation to either oral or silent reading fluency, which was not as expected. The ability of visual rapid temporal processing was measured by the phantom contour paradigm of Sperling et al. (2003, 2006). To our knowledge, there has been no report using this paradigm to explore the relationship between visual rapid temporal processing and Chinese reading fluency in skilled readers. A previous study on Chinese children found that their performance in the phantom contour paradigm was correlated with oral reading fluency in single-character level (Xiao et al., 2014). The current finding was inconsistent with the previous research of Xiao et al. (2014), and this inconsistency may be explained by the developmental difference of the participants. Some researchers had pointed out that maturation can affect the relationship between visual rapid temporal processing and fluent naming (Englund and Palomares, 2012). It has been found that the visual rapid temporal processing was related to reading fluency in English among 5-to-15 year-old learners, but this correlation was not significant once participants’ age was controlled (Englund and Palomares, 2012).

Primary school students in China learn Mandarin mainly through Pinyin, an alphabetic phonetic system used to bridge the gap between speech and the written form of Chinese characters for beginning readers (Siok and Fletcher, 2001). It has been found that Pinyin exhibited the similar mechanism of the orthographic-to-phonological mapping as in alphabetic writing systems (Wang et al., 2005). The orthographic-to-phonological mapping is a key element of reading fluency (Norton and Wolf, 2012; Eberhard-Moscicka et al., 2014; Hakvoort et al., 2015). The visual rapid temporal processing was hypothesized to be crucially involved in the mapping between spelling and sound as it requires the visual engagement and disengagement of each sub-lexical unit (Gori et al., 2014; Ruffino et al., 2014). Thus, the relationship between visual rapid temporal processing and reading fluency may be mediated by the role of the visual skill in the sub-lexical mapping between orthography and phonology. With the increase in reading experience, the utilization of Pinyin in reading procedure would be gradually diminished (Huang and Hanley, 1995), and the orthographic characters can be map onto speech sounds directly without the mediation of Pinyin (Tan et al., 2005). Thus, the developmental changes between developing and proficient readers may reduce the involvement of visual rapid temporal processing in reading fluency.

The lacking of relationship between visual rapid temporal processing and reading fluency was inconsistent with previous research in alphabetic languages. Sperling et al. (2006) reported that English adults’ performance in the phantom contour paradigm was related to oral reading fluency in single word level. The inconsistent result can be explained by the difference in which orthographic input are linked to speech sounds between alphabetic and non-alphabetic languages. For skilled readers in English, the visual symbols of letters map onto the sound units based on the grapheme-phoneme conversion rules (Tan et al., 2005). The visual rapid temporal processing has been found to play a role in the orthographic-to-phonological mapping in the sublexical route (Gori et al., 2014; Ruffino et al., 2014), which would explain the relationship between visual rapid temporal processing and reading fluency of English. In contrast, the orthographic characters map onto speech sounds for Chinese skilled readers through an addressed way (i.e., a global mapping between a orthographic character to its pronunciation, and it is different from letter-by-letter spelling in English; Tan et al., 2005). In this circumstance, visual rapid temporal processing may have little influence on the mapping between orthography and phonology in Chinese adults and this may explain the absence of relationship between visual rapid temporal processing and reading fluency in skilled readers.

Although, we found that the visual rapid simultaneous processing was correlated with both oral and silent reading fluency, the comparison of the correlation coefficients revealed a closer relationship between the visual rapid simultaneous processing and silent reading. Result of the regression analysis showed that visual rapid simultaneous processing made a significant contribution to silent reading fluency but not oral reading fluency. The present study used the visual attention span as an index of the rapid simultaneous processing. Thus far, no studies have investigated the relationship between visual attention span and read fluency in Chinese directly. However, previous neuroimaging studies showed that the activation of the bilateral parietal areas functioning on visuospatial attention processing was associated with superior reading fluency in Chinese (Siok et al., 2009; Qian et al., 2015, 2016). This reveals the relationship between visuospatial attention and Chinese reading fluency, to some extent. Moreover, Wang et al. (2015) have found that silent reading of Chinese sentences would induce activation in the middle temporal gyrus which is thought to be important for the direct mapping of orthography to semantics. Based on this finding, it can be proposed that Chinese readers’ visual attention span may have an impact on the parallel processing of multiple orthographic units of Chinese characters which in turn may affect the efficiency of their sentence comprehension ability during the silent reading task.

The present finding was consistent with previous research in alphabetic languages (van den Boer et al., 2014) in which they also found that children’s visual attention span showed a significant unique contribution to their silent reading fluency but not oral reading fluency. Given that Chinese is an ideographic language in which visual-semantic processing plays a special role (Wang et al., 2003; Yan et al., 2010), the visual attention span might be more closely related to Chinese reading fluency in silent compared to oral reading mode. Future studies is required to examine whether the relationship between visual rapid simultaneous processing and reading fluency is similar across different language systems.

The current study showed that visual rapid simultaneous processing made a unique contribution to silent reading fluency but not oral reading fluency. This highlights the differences in the underlying skills of reading fluency across the two modes in the general cognition level. The connectionist multi-trace model of word reading (Ans et al., 1998) postulates that reading relies on two reading procedures, a global and an analytic one. In global reading mode, the visual attention window extends over the whole sequence of the input string whereas the visual attention window narrows down to focus attention successively on different parts of the input when reading in analytic mode (Bosse et al., 2007). Based on this model, global processing typically requires a larger visual attention span than analytic processing (Bosse et al., 2007). Wang et al. (2015) has showed that silent reading mainly relies on the global orthographic-to-semantic mapping. In contrast, oral reading fluency has been reported to be involved in the orthographic-to-phonological mapping. This may explain the absence of relationship between visual attention span and oral reading fluency in the current study. If this account is broadly correct, then it can be suggested that reading fluency in the silent mode may rely on the global reading procedure while reading fluency in the oral mode may rely on the analytic reading procedure.

In summary, the current study did not find a significant relationship between the visual rapid temporal processing and Chinese reading fluency in either silent or oral mode. This illustrates the relationship between visual rapid temporal processing and reading fluency depends upon the characteristics of the language. The visual rapid simultaneous processing demonstrated a unique contribution to silent reading fluency but not to oral reading fluency. This may imply that the underlying mechanism between oral and silent reading fluency is different in the beginning of the basic visual coding.

## Ethics Statement

The study was approved by the institutional review board of the Department of Psychology, Capital Normal University, Beijing, China. Written consent was obtained from each participant prior to the experiment. The participants were recruited by the Internet.

## Author Contributions

JZ designed and wrote the manuscript. RK revised the manuscript critically for the intellectual and grammatical content. ML performed the experiment and analyzed data. HL, CH implemented the computerized experiment.

## Conflict of Interest Statement

The authors declare that the research was conducted in the absence of any commercial or financial relationships that could be construed as a potential conflict of interest.
